# Outcome of bone defect reconstruction with clavicle bone cement prosthesis after tumor resection: a case series study

**DOI:** 10.1186/1471-2474-15-183

**Published:** 2014-05-29

**Authors:** Bin Lin, Yong He, Yang Xu, Mo Sha

**Affiliations:** 1 Department of Orthopedics, the 175th Hospital of PLA, Southeast Hospital of Xiamen University, Zhang Zhou, Fu Jian, China

**Keywords:** Clavicle malignancy, Claviculectomy, Bone defect, Bone cement prosthesis

## Abstract

**Background:**

To investigate the short and medium term outcomes of bone defect reconstruction with bone cement prosthesis after clavicle malignancies resection.

**Methods:**

A total of 5 clavicular malignancy patients experienced bone cement prosthesis reconstruction after subtotal claviculectomy were enrolled the study from January 2005 to May 2012. Musculoskeletal Tumor Society score (MSTS), Visual Analogue scale (VAS) and American Shoulder and Elbow Surgeons shoulder outcome score (ASES) were adopted for assessment.

**Results:**

The mean follow-up period was 25.8 months. All patients were performed bone cement defect reconstruction after claviculectomy. In which, 3 cases showed disease-free and other 2 cases were alive with sickness. The average Musculoskeletal Tumor Society score was 85.40% ± 5.68%(77%-90%), Visual Analogue Scale was 1.40 ± 0.55 (1–2) and American Shoulder and Elbow Surgeons Shoulder Outcome Score was 92.40 ± 3.29(87–96).

**Conclusions:**

Adoption of clavicle bone cement prosthesis for bone defect reconstruction after tumor resection can maintain the contour of shoulder and reduce the complications ascribe to the claviculectomy. It is an effective and feasible therapeutic procedure in clinical setting.

## Background

The clavicle is a rare site of bone tumors growth [1]. The literature referring to the clavicle is devoid, usually is just involving a case report and a few patients [1-5]. Orthopedic oncologists consequently have only insufficient experience in management of clavicle malignancies. Currently, claviculectomy is still a main surgical method for the treatment of this disears even though it was performed as early as 1912 by Gurd [6]. Unfortunately, the oncologic outcome of this operation is far away from the satisfaction [5,7]. Rubright J et al. [8] concluded that with time, gradually lose some compensatory ability as evidenced by deteriorating limb-specific, patient-centered outcome measures, diminished strength in certain planes of shoulder motion, and scapular dyskinesis at long-term follow-up. With the great improvement of diagnostic and therapeutic techniques recently, patients with a musculoskeletal malignant tumor are expected to have longer survival period. However, the type of claviculectomy continues maintaining a high incidence of surgical complications and related side effects, such as vascular injury, never damage and infection, etc. [9-11]. In order to decline the high incidence of surgical complications, some surgeons suggested a new concept of defect reconstruction after claviculectomy with the materials of autogenous bone [12-15] or allograft bone [14,16]. These treatments can protect the subclavian vessels and brachial plexus from operative damage, restore the shape of shoulder and relieve the pain. The method we described has performed by Vartanian et al. [17], they reconstructed the bone defect after metastatic tumor resection onto the medial third of clavicle, extending of the manubrium and the anterior portion of the first rib with bone cement composited prosthesis, named “Oklahoma prosthesis”. The results showed that this method was a simply and quick technique to effectively stabilize the acromioclavicular joint while preserving the appearance of clavicle and shoulder girdle. To the best of our knowledge, however, the recovery of shoulder function and the oncologic outcome after reconstruction have not been reported so far. Therefore, the effects of bone reconstruction after claviculectomy are not justified. The aim of this study is to report the outcomes of the shoulder function and oncologic results in 5 patients who underwent bone defect reconstruction with bone cement prosthesis after clavicle malignancies resection.

## Methods

### Patients

**Figure 1:**
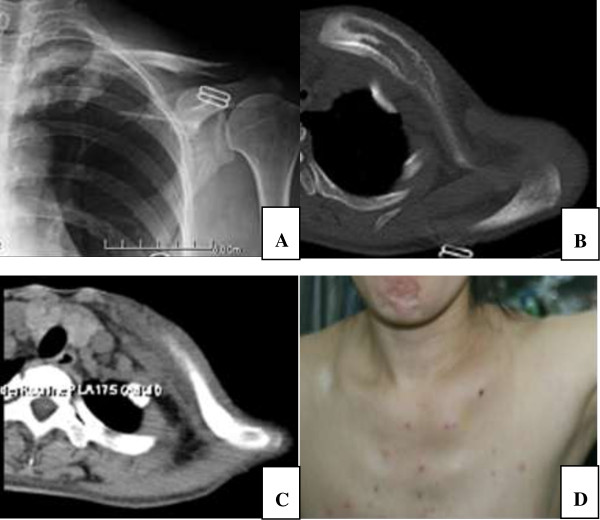
**A 31 years female patient with bone lymphoma of left clavicle.**Iconography shows bone expansion and soluble bony destruction. The main symptoms are swelling and pain. **A**. Preoperative X-film shows osteolytic destruction of left clavicle. **B**, **C**. Preoperative CT scan show the range of lesion, no subclavian vein compression. **D**. Outside view of shoulder, main sign is swelling.

Five patients with unilateral clavicle malignancies during the period of 2005–2012 were included the study. Patients were three males and two females with an average age of 37.8 years (19–62 years). The types of malignant tumors among the 5 patients were 2 Ewing sarcomas, 1 osteosarcoma, 1 bone lymphoma and 1 chondrosarcoma. The tumors were located at the medial third of clavicle in 3 cases, at the middle third in 1 case and at the lateral third in 1 case respectively (Table 1). The majority of the patients presented the symptoms of swelling (Figure 1) with or without pain, and one patient presented pain and stiffness of shoulder. None of the patients had neurovascular deficit, whereas all showed the restriction of shoulder movement.

**Table 1:** Clinical data of all patents

**Case**	**Sex**	**Age(years)**	**Diagnosis**	**Location**	**Treatment methods**	**Reconstruction**	**Metastasis**	**Recurrence**	**Oncologic results**	**Follow up(mon)**
1	M	19	Ewing sarcoma	Medial third	Subtotal resection + radiotherapy	Bone cement	None	None	NED	11
2	M	34	Osteosarcoma	Lateral third	Subtotal resection + chemotherapy	Bone cement	None	None	AWD	24
3	Fe	43	Ewing sarcoma	Medial third	Subtotal resection + radiotherapy	Bone cement	None	None	NED	16
4	M	62	Chondrosarcoma	Medial third	Subtotal resection	Bone cement	None	None	NED	58
5	Fe	31	Bone lymphoma	Middle third	Subtotal resection + chemotherapy	Bone cement	None	None	AWD	20

*NED*, no evidence of disease.

*AWD*, alive with disease.

The preoperative average VAS score was 8.00 ± 1.41(6–9), MSTS was 61.80% ± 8.53%(49%-71%) and ASES was 42.40 ± 9.02(33–52) (Table 2).

**Table 2:** Outcomes

	MSTS (score %)*		ASES(score)*		VAS(score)*		**Complications**	**Outcome of reconstruction**
**Case**	**Preop.**	**Postop.**	**Preop.**	**Postop.**	**Preop.**	**Postop.**		
1	58	77	33	87	9	1	None	Intact
2	49	82	52	93	9	2	None	Intact
3	65	89	34	93	9	2	None	Intact
4	66	89	51	96	6	1	None	Intact
5	71	90	42	93	7	1	None	Intact

*Paired-Samples *T* test(P < 0.05).

*MSTS*, Musculoskeletal Tumor Society score;

*ASES*, American Shoulder and Elbow Surgeons shoulder outcome score;

*VAS*, Visual Analogue scale.

The Study was approved by the 175th PLA hospital Ethics Committee. Every patients approved publish their information, images and legends. Written informed consent for participation in the study was obtained from every patient.

### Preoperative preparation

Preoperative computed tomographic scan (CT and MRI) revealed the scope of lesion and the compression of subclavian vein (Figure 1). All patients were administrated cefuroxime intravenously before surgery to prevent the infection (ESSETI FARMACEUTICI S.R.L.).

### Surgical procedure

**Figure 2:**
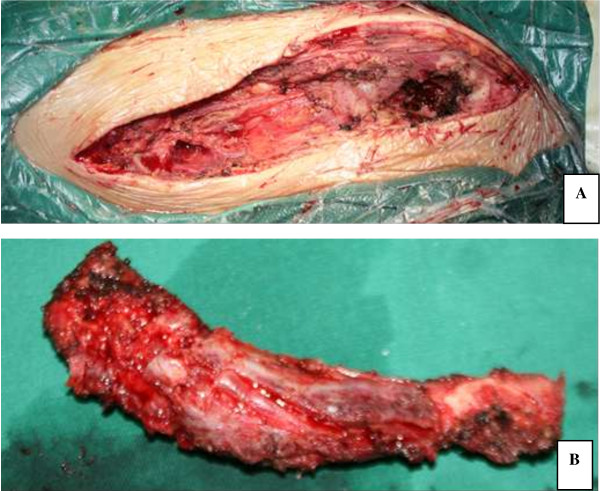
**Intraoperative photograph. A**. Resection of clavicle length **B**. Resected specimen.

**Figure 3:**
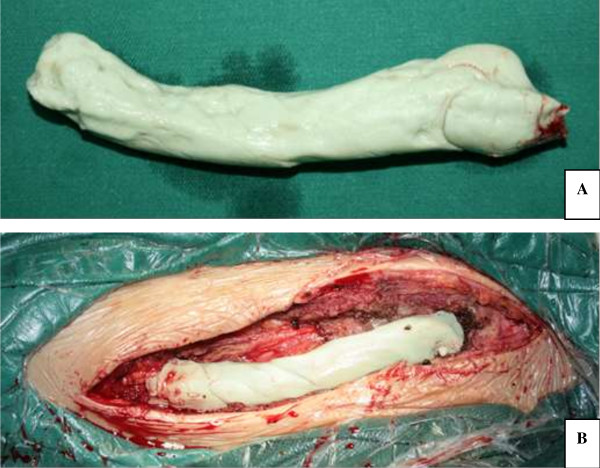
**Intraoperative bone cement prosthesis was made by surgeons. A**. Size of bone cement prosthesis based on the preoperative CT scan **B**. Reconstructed bone defect with bone cement prosthesis.

The clavicle was exposed through a curvilinear extensile approach following the bone outline from the sternoclavicular to the acromioclavicular joint. The musculofascial envelope and periosteum were incised. Circumferential blunt dissection was carefully operated and the clavicle was gently disarticulated and elevated out of the bed (Figure 2). A standby vascular surgeon was required throughout the surgical process. After removal of the clavicle, the bone defect was reconstructed with bone cement with antibiotics (PLUS Endoprothetik AG) prosthesis (Figure 3). The subcutaneous tissue was closed in layers over the prosthesis with placing two superficial drains. No skin was resected and the wound was primarily closed.

### Postoperative management

The patients need wear an arm sling for four weeks after surgery. The passive range of motion was allowed at three weeks, and the active range of motion started at six weeks. Theraband and pilates exercises commenced at two months in all patients and progressed gradually depend on the tolerance of patient. The motion of shoulder shrugs and wall push-ups were encouraged. The formal physical therapy regime was not employed and all patients were maintained on a home exercise program for a minimum of six months after surgery. This program included thrice-weekly maintenance range of motion and resistance exercises for all shoulder girdle muscles. Operative patients were assessed with the Visual Analogue Scale (VAS), Musculoskeletal Tumour Society Score (MSTS) [18] and the Functional Evaluation Form recommended by the American Shoulder and Elbow Surgeons (ASES) shoulder outcome score [19]. All statistical analyses were performed using the SPSS 18.0.0 statistical package (SPSS, Inc., Chicago, IL, USA), and p value of less than 0.5 was accepted as significant. The data are presented as mean ± standard deviation. Statistical differences in MSTS, ASES and VAS before and after surgery were compared using Paired-Sample *T* Test.

## Results

**Figure 4:**
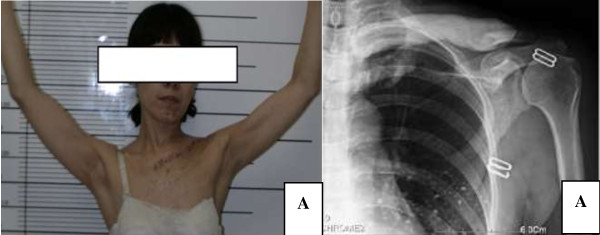
**Shoulder outline and X-ray of postoperation. A**: Post-operative shoulder outline and functional display. Patient has an aesthetic shape and favorable function **B**. Radiograph of post bone cement reconstruction shows a fine prosthesis location.

Demographic information of five patients was shown in Table 1. All patents were executed subtotal claviculectomy and bone defect cement reconstruction, in which 2 patients received chemotherapy and other 3 patients experienced radiotherapy. The average follow-up period was 25.80 ± 18.63 months (11–58 months). After surgery, none of the patients demonstrated neurological deficit and vessels injury, as well as local recurrence and metastases of the tumors. Of all 5 patients, 3 cases showed disease-free, whereas other 2 were alive with sickness. No infection and rejection reaction were found and all prosthesis were intact and the shape of shoulders was acceptable without significant functional deficit (Figure 4). The average Musculoskeletal Tumor Society score was 85.40% ± 5.68%(77%-90%), Visual Analogue scale was 1.40 ± 0.55(1–2) and American Shoulder and Elbow Surgeons shoulder outcome score was 92.40 ± 3.29 (87–96), all parameters were of statistical differences significantly compared with those before surgery (p < 0.05) (Table 2).

## Discussion

The incidence of primary clavicle tumors occupied 0.45% to 1.01% within all bone tumors [20,21]. In the most cases, clavicular tumor is a malignant one. Radical surgery to remove the tumor is a major therapeutic option even if it is usually not to be accomplished easily. Rossi B et al. [5] reported 4 patients with clavicle malignant tumors after claviculectomy, who did not have the local tumor recurrence and the average MSTS score reached to 86.6% and the mean Constant–Murley score arrived to 80 with no functional impairment. In our study, the average MSTS score of the patients was 85.40% ± 5.68%. Krishnan et al. [22] performed a total claviculectomy as a salvage procedure for 6 patents with clavicle malignancies, resulted in the restoration of partial shoulder functions, led to the minimal strength deficits and provided durable pain relief. Ledger [23] and Green et al. [24] considered that claviculectomy was an acceptable approach in the treatment of clavicle malignant tumor. However, the procedure was often accompanied by a high occurrence of a variety of complications such as vascular injury, brachial plexus damage, shoulder instability and disturbed pain. After clavicle resection, it might change the scapula’s motion tract, induce the uneven distribution of shoulder stress and shoulder instability, and result in chronic damage of shoulder joints and shoulder arthritis, thereby gave rise to joint pain, weakness, motion restriction, oblique shoulder and drop shoulder. Likely, the clavicle bone defect can provoke the vascular injury and brachial plexus damage because of the diminishing of protective effects. Vartanian et al. [17] recommend that the purposes of clavicle reconstruction after claviculectomy were to recover the shape of shoulder, restore the normal motion track of upper limb, protect to neurovascular bundle, maintain the shoulder function and make the upper limb attached to truncal bone indirectly, so as to reduce the incidence of complications after clavicle excision.

Currently, the major materials of bone defect repairmen after clavicle resection include autogenous rib, fibula [12-15] and allograft bone [14,16]. Reconstruction with autologous bone can achieve the aims of favorable histocompatibility and satisfying integration of bone and soft tissue, which can last long term for good stability. However, this method would stimulate the growth of new trauma, develop a series of complications at other location such as bone defect and infection, and is difficult to repair the shape of shoulder. As to the large segment bone defect involving joints, it is difficult to attain the anatomic recovery, so as to increase the risk of joint dislocation. On the contrary, allograft bone can recover a nice shoulder contour and improve the standard of daily life with long term favorable outcomes. However, the potential complications such as infection, nonunion and inferior integration of bone and soft tissue limited its extensive application in clinical setting. Momberger et al. [14] reported 2 patients with clavicle bone defect after clavicle trauma were received reconstruction with allograft bone, unfortunately the allograft was taken out and replaced by vascularized fibula grafts finally due to severe shoulder pain and nonunion.

To our knowledge, the literatures regarding the bone defect reconstruction with cement prosthesis after clavicle tumor resection are few. Vartanian et al. [17] reported a patient with clavicle metastatic from renal cell carcinoma was performed an En bloc clavicle excision and treated bone defect with bone cement after resection. In this procedure, no skin was resected and the wound was primarily closed. They found that the postoperative outcome displaying the normal outline of the acromioclavicular joint with good motion range at the shoulder. However, it is only the report of this case and lack of enough assessment to the median and long term functional outcome of the shoulder.

This study described a series of five patients with clavicle malignant tumor who were performed subtotal clavicle resection and clavicle bone defect reconstruction with bone cement prosthesis, the contours of shoulder were acceptable by patients and did not occur relative complications such as infection and rejection reaction. At the end of follow-up period, 3 patients were disease-free and 2 were alive with sickness. The postoperative mean MSTS score was 85.40% ± 5.68%, ASES was 92.40 ± 3.29 and VAS score was 1.40 ± 0.55, all of them demonstrated significant statistical differences compared those before operation respectively (P < 0.05). These outcomes indicated that surgical management by bone cement prosthesis reconstruction for clavicle bone defect after excision is an favorable method in restoration of the shoulder shape and recovery of the function of shoulder joint effectively. In this study, the American Shoulder and Elbow Surgeons shoulder outcome scores of all patients after surgery were significant improved compared with those before surgery. The tumor prognosis was well and did not find relative complications such as rejection and infection. It protected the subclavian vessels and brachial plexus, so as to avoid the secondary damage. This procedure can maintain a supportive function of shoulder and play a little role on heavy overhead work of shoulder joint, however, it will not reach the osseous fusion after reconstruction with bone cement prosthesis and may develop pseudarthrosis, which will negatively affect the suspension of shoulder joint and result in mild pain long after operation and acromioclavicular joint instability. However, these symptoms are not so serious and can be tolerated by most patients.

Bone cement prosthesis for reconstruction of bone defect after clavicle malignancies resection is a simple, feasible and effective method. This procedure can introduce the beneficial effects to the patients in short and median term. However, its long term outcomes are controversial. Many researchers suggest to select the alternative reconstruction method for the patients with long life expectancy. On the side, our surgical method will provoke the undesirable integration of bone and soft tissue. Since the less number of enrolled patients, the evidence grade of this study was lower. For further verifying the long term effect of this method, the large scale, multicenter and randomized controlled stuies are required. Although the outcomes of claviculectomy and reconstruction are satisfied, the radiotherapy and chemotherapy after operation are necessary. It is likely the reasonably functional exercise of shoulder is essential for the patient.

## Conclusions

Clavicular malignancies are rare types of tumor with poor prognosis. Claviculectomy alone is probably suitable for local tumor control only. This study confirmed that total or subtotal clavicle excision with bone cement reconstruction after malignancies surgery is rarely associated with the significant shoulder function loss. However, we recommend to take the other reconstruction materials to the patients with long life expectancy.

## Competing interests

All authors declare that they have no competing interests.

## Authors’ contributions

YH and YX performed statistical analyses, drafted the manuscript and critically reviewed the final manuscript. MS and BL performed database searches, drafted the manuscript and elaborated the figure. All authors read and approved the final manuscript.

## Pre-publication history

The pre-publication history for this paper can be accessed here:

http://www.biomedcentral.com/1471-2474/15/183/prepub
